# On two forgotten European species of Coleoptera

**DOI:** 10.3897/zookeys.749.24275

**Published:** 2018-04-10

**Authors:** Yves Bousquet

**Affiliations:** 1 Gatineau, Quebec, Canada

**Keywords:** Coleoptera, beetles, Carabidae, Oedemeridae, Ljubljana, Ferdinand Joseph Schmidt

## Abstract

Two Coleoptera species described by Ferdinand Joseph Schmidt in 1834 have been forgotten. One, *Chlaenius carniolicus*, is placed here in synonymy with Chlaenius (Chlaenites) spoliatus (Rossi, 1792), while the other, *Oedemera lippichii*, is synonymized with Nacerdes (Xanthochroa) carniolica carniolica Gistel, 1834 (**new synonymies**). *Chlaenius carniolicus* Gistel, 1834, a primary homonym of *C. carniolicus* Schmidt, 1834 which has been forgotten too in the literature, is also placed in synonymy with *Chlaenius spoliatus* (**new synonym**).

## Introduction

In 1834, Franz Wilhelm Lippich [1799–1845], a Slovenian physician, published a book providing various information about Ljubljana, the capital of Slovenia, which at the time was part of the Austrian Empire. Part of the information relates to the natural history of the city (pp. 43–66) and includes among others a list of the insects (pp. 58–66). The Coleoptera are on pages 60–66 and two new species are described in footnotes: *Chlaenius carniolicus* (p. 60) and *Oedemera lippichii* (p. 62). The descriptions of both new species were provided by Ferdinand Joseph Schmidt [1791–1878], an Austro-Hungarian entomologist and businessman, to whom the species names should be attributed.

As far as is known, these two species have not been recorded subsequently. For example, *Chlaenius carniolicus* is not listed in the Chlaeniini section for the first (Kirschenhofer 2003) and second (Kirschenhofer 2017) editions of Volume 1 of the *Catalogue of Palaearctic Coleoptera* and *Oedemera lippichii* is not listed in the Oedemeridae section (Śvihla 2008) in Volume 5 of the same series. Neither name is recorded in Sherborn’s *Index Animalium* or in Gemminger and Harold’s *Catalogus Coleopterorum*.

The descriptions of the two species are provided here, following by my translation.

“Eine neue Species, über welche mir Hr. Schmidt Folgendes mittheilt: *Chlaenius carniolicus*, (mihi) viridi-aeneus, thorace subcordato-ruguloso, antennis pallidis, elytris glabris, subtiliter punctato-striatis, margine flavis, pedibus rufo-piceis. - Hat einige Aehnlichkeit mit dem *Chlaenius spoliatus*, ist jedoch 1 bis 1 1/2 Linie länger und verhältnissmässig auch breiter. Der Kopf ist stark gerunzelt, eben so der mit einer tiefen Mittel- und zwei Seitenfurchen versehene flache Halsschild, worauf unter dem Oberrande zwei Eindrucke sich befinden. Fühler und Fressspitzen sind braungelb, die Füsse pechbraun. Die Oberseite des ganzen Käfers ist metallgrün, die Flügeldecken sind kahl, seicht gefurcht, und mit feinen Puncten in den Streisen besetzt. An den Ufern des Gruber’schen Kanals hinter dem Laibacher Schlossberge bisher allein aufgefunden, sehr selten.” [A new species for which Schmidt wrote the following: *Chlaenius carniolicus*, (mihi) greenish bronze, pronotum subcordate and rugose, antennae pale, elytra glabrous, slightly punctate and striate, margins yellowish, legs rufopiceus. The species has some resemblance to *Chlaenius spoliatus*, but is 1 to 1½ lines longer and comparatively broader. The head is heavily wrinkled, as well as the pronotum which is flat and has a deep median and two lateral furrows, as well as two impressions at the anterior margin. Antennae and extremities of palps brownish, the legs pitch-brown. The dorsum of the beetle is metallic green, the elytra bare, the striae shallowly and finely punctured. A single specimen found on the banks of the Gruber’s canal behind Ljubljana castle hill.]

“*Oedemera lippichii* (Schmidt). *Oedemera* thorace lato nigro-marginato, elytris fusco-viridibus striatis, pedibus flavis. - Etwas grösser als *Oedemera annulata*. Hat einen gelben Kopf und Halsschild, schwarze Augen, ziemlich breiten, schwarz gerandeten Thorax, das Schildchen ist gelb, eben so die Füsse, die Flügeldecken stahlgrün, mit erhabenen Streifen. Ich habe von dieser Art in sechs Jahren blos drei Individuen, auf Dolden des Krimberges vorkommend, gefunden.” [*Oedemera* with large black pronotal margins, elytra dark green with striae, legs yellowish. Somewhat bigger than *Oedemera annulata*. Head and pronotum yellowish, eyes black, relatively large, pronotum margins black, the disc yellow like the legs, the elytra steel-green with uneven striae. In six years I have found only three specimens on umbellifers on Mount Krim].

Based on the description, particularly the coloration, *Chlaenius carniolicus* is very likely a synonym of C. (Chlaenites) spoliatus (Rossi, 1792). The same year Gistel (1834: 149) also described a new species under the name *Chlaenius carniolicus* from Laibach (= Ljubljana), which has likewise been forgotten in the literature. The few descriptive words provided by Gistel suggest that his *C. carniolicus* is the same as that described by Schmidt. In fact, it is possible that both descriptions are based on the same specimen. In his paper, Gistel (1834: 147) mentioned under *Geocharis thoracica* “Museum Dr. Schmidt Labaci” indicating that he probably had access to Schmidt’s collection or was in possession of some of Schmidt’s specimens. Gistel’s (1834) work was issued in the third part of the first volume of his journal *Faunus*, which was published by 16 August 1834 as noted in *Die Bayer’sche Landbötin* (No 98: 802); it was also noted in the 31 August 1834 issue of *Bibliographie von Deutschland* (vol 9: 195). I have been unable to find a more specific date of publication for Lippich’s work in 1834. As it stands now, *Chlaenius carniolicus* Schmidt, 1834 is a junior primary homonym of *Chlaenius carniolicus* Gistel, 1834.

Gistel (1834: 150) described *Oedemera carniolica* (p. 150) also from Mount Krim [“Krimmberge in Krain”] which is currently considered a valid species in the subgenus Xanthochroa W.L.E. Schmidt, 1844 of the genus *Nacerdes* Dejean, 1834 (Śvihla 2008: 363). The color described by Schmidt for *O. lippichii* clearly suggests that both taxa are identical. It is interesting to note that Schmidt (1846: 36) himself listed “*Necydalis Lippichii*. Kunze in litt.” as synonym of *Xanthochroa carniolica* Gistel.

According to Horn et al. (1990: 350), Schmidt’s collection was acquired in 1935 by the Slovenian Museum of Natural History in Ljubljana [formely known as *Krainer Landesmuseum „Rudolfinum*” in honor of the Crown Prince Rudolph (1858-1889)]. Upon my request Dr. Tomi Trilar of the Slovenian Museum checked Schmidt’s collection but was unable to find any specimens under the names *Chlaenius carniolicus* and *Oedemera lippichii*. Even if the type specimens cannot be study at this time, I believe the original descriptions clearly suggest that *Chlaenius carniolicus* Gistel, 1834 and *Chlaenius carniolicus* Schmidt, 1834 are junior synonyms of *Chlaenius spoliatus* (Rossi, 1792) and *Oedemera lippichii* a junior synonym of *Nacerdes carniolica carniolica* Gistel, 1834 (**new synonymies**).
